# Screening Social Anxiety in Adolescents Through the Eyes of Their Carers

**DOI:** 10.3389/fpsyg.2021.769006

**Published:** 2021-12-02

**Authors:** Luis-Joaquin Garcia-Lopez, Lourdes Espinosa-Fernandez, Jose-Antonio Muela-Martinez, Jose Antonio Piqueras

**Affiliations:** ^1^Department of Psychology, University of Jaén, Jaén, Spain; ^2^Department of Health Psychology, Miguel Hernández University, Elche, Spain

**Keywords:** adolescence, anxiety, assessment, carers, online, psychometrics, social anxiety disorder

## Abstract

Despite the availability of efficacious treatment and screening protocols, social anxiety disorder (SAD) in adolescents is considerably under-detected and undertreated. Our main study objective was to examine a brief, valid, and reliable social anxiety measure already tested to serve as self-report child measure but administered *via* Internet aimed at listening to the ability of his or her parent to identify social anxiety symptomatology in his or her child. This parent version could be used as a complementary measure to avoid his or her overestimation of children of social anxiety symptomatology using traditional self-reported measures. We examined the psychometric properties of brief and valid social anxiety measure in their parent format and administered *via* the Internet. The sample included 179 parents/legal guardians of adolescents (67% girls) with a clinical diagnosis of SAD (mean age: 14.27; *SD* = 1.33). Findings revealed good factor structure, internal consistency, and construct validity. Data support a single, strength-based factor on the SPAIB-P, being structure largely invariant across age and gender. The limited number of adolescents with a performance-only specifier prevented examining the utility of scale to screen for this recently established specifier. It is crucial to evaluate if these results generalize to different cultures and community samples. The findings suggest that the SPAIB-P evidences performance comparable with child-reported measure. Parents can be reliable reports of the social anxiety symptomatology of the adolescent. The SPAIB-P may be useful for identifying clinically disturbed socially anxious adolescents.

## Introduction

Social anxiety disorder is one of the most prevalent childhood psychiatric disorders and tends to be a chronic, stable condition that severely disrupts social and academic functioning (Lijster et al., 2018; Chiu et al., 2021). Despite this, social anxiety disorder (SAD) is under-recognized and undertreated (Jefferies and Ungar, 2020). Without accurate early detection of SAD, youth cannot access to evidence-based interventions (Garcia-Lopez et al., 2015). Brief social anxiety measures can screen for at-risk anxious adolescents (Fuentes-Rodriguez et al., 2017). Parents are in a key position to identify and screen mental health problems early and to provide a link to appropriate services (Mautone et al., 2020). A major advantage of parent-report measures is that they draw on the extensive knowledge parents have about their children and offer a more comprehensive perspective of SAD symptomatology (Garcia-Lopez et al., 2005). Unfortunately, the role of parents as informants of screening for emotional problems in their children has been ignored. This may be partially due to the large discrepancies in social anxiety symptomatology found between parents and adolescents (Becker-Haimes et al., 2018; Deros et al., 2018). Despite this, preliminary findings in early 2000s suggested that parents can be reliable reports of the social anxiety symptomatology of the adolescent (La Greca et al., 2001; Perez et al., 2001). In addition, some authors have argued that, given that adolescents with social anxiety disorder often try to make a good impression to mental health providers, the inclusion as informants of parents, teachers, or significant others might contribute to the correct identification of subjects (Garcia-Lopez et al., 2010). Despite this, no paper has been published to address the psychometric properties of the parent version of a well-established child-report social anxiety measure. Thus, our main study objective was to examine psychometric properties of the parent-report version of Social Phobia and Anxiety Inventory-Brief form (SPAI-B; Garcia-Lopez et al., 2008) in a clinical sample of adolescents with SAD.

First, this paper was aimed at testing whether SPAI-B/P would retain the good psychometric properties evidenced by the original scale in an online format (factor structure, internal consistency, and construct validity) (Garcia-Lopez et al., 2008, 2014, 2015, 2018; Piqueras et al., 2012; Vieira et al., 2013; Moran et al., 2018). Second, it was evaluated whether parent version was invariant for gender and age of his or her child.

To the best of our knowledge, this was the first paper analyzing the role of parents as informants of social anxiety symptomatology of their adolescents using a parent-report measure stemming from a well-established child-reported scale.

## Materials and Methods

### Participants

The participants were 179 adolescents (67% girls) in Grades 8 to 12 (aged 12–18 years; *M* = 14.27; *SD* = 1.33) and parents or legal guardians. The ethnicity of the adolescents was 76% European-Spaniards, 19% Hispanic-American, and 5% Central-European. These data are consistent with the social reality in the country according to INE data (National Statistics Institute, 2020). To code the socioeconomic status of the participating subjects, the profession held by the most socioeconomically advantaged parent at the time of study was selected. Socioeconomic status for this sample was predominantly middle class, as categorized by Hollingshead Social Class (Hollingshead, 1975; Level I: 6.3%; Level II: 5.%; Level III: 16.7%; Level IV: 21%; Level V: 13.5%).

### Measures

Adolescents completed the SPAI-B. Parents or legal guardians completed the SPAIB-Parent form and SAS-A/Parent form. For a review of the psychometric properties of the instruments listed below, see Garcia-Lopez et al. (2015).

Social Anxiety Scale for Adolescents (SAS-A; La Greca and Lopez, 1998). This questionnaire contains 18 items (plus four filler items) and includes three subscales: Fear of Negative Evaluation (FNE; eight items), Social Avoidance and Distress specific to new situations or unfamiliar peers (SAD-New; six items), and Social Avoidance and Distress that is experienced more generally in the company of peers (SAD-General; four items). Items are rated on a five-point Likert scale (1-5) and summed across relevant items to obtain total SAS-A scores and scores for each of the three subscales. A parent version (SAS-P) has also been developed, with promising preliminary results (La Greca et al., 2001; Perez et al., 2001).

The Social Phobia and Anxiety Inventory, Brief form (SPAI-B; Garcia-Lopez et al., 2008) consists of 16 items using a five-point Likert scale (1-5). It assesses the cognitive, somatic, and behavioral symptoms (triple-response system) and captures interactional and performance-provoking socially anxious situations. However, the brief form is different from the original form in terms of the Likert scale format used, the number of items, and avoidance of heterocentric language. Items 15 and 16 are comprised of subitems related to somatic and cognitive symptoms; hence, item 15 is scored as the average of four subitems, and item 16 as the average of five subitems. Therefore, decimals can be obtained. The SPAI-B score is the sum of item ratings minus 16. As a result, a total score can be computed (range: 0–64). The SPAI-B Parent version includes the same items but reworded to reflect views of a parent on the symptomatology of his or her child.

The Anxiety Disorders Interview Schedule for DSM-5–Child and Parent Versions (ADIS5-C/P); Albano and Silverman, in press) assesses anxiety disorders in youth aged six to 17 years and is organized according to anxiety disorders included in the Diagnostic and Statistical Manual of Mental Disorders, Fifth Edition [(DSM-5); American Psychological Association [APA], 2013]. The ADIS-5-C/P consists of comparable yet separate child and parent interviews. A Spanish translation by authors was administered subsequent to obtaining approval of Oxford University Press for the ADIS5-C/P to be used for research purposes.

### Procedure

The study was approved by the School District and the University Research Ethics Committee in compliance with the Code of Ethics of the World Medical Association (Declaration of Helsinki of 1975, revised in 2013) and the Charter of Fundamental Rights of the European Union.

Adolescents were screened for social anxiety in their schools. Those who screened positive as being at risk for SAD and a percentage of those who screened negative for SAD were invited to participate in a comprehensive, individualized assessment (ADIS5-C/P, see above) to determine the presence or absence of a clinical diagnosis of SAD. After the diagnostic interview was administered, 227 (6% of the original sample, consistent with prevalence reported by other studies (e.g., Garcia-Lopez et al., 2009, 2014, 2015) were diagnosed with a clinical diagnosis of social anxiety disorder (15% criteria for the performance-only specifier). About 79% of parents (*N* = 179 out of 227) also participated in the study and signed the consent forms online. Completion of the measures took approximately 20 min.

### Statistical Analyses

Cronbach’s alpha, the greatest lower bound (glb), and the omega total coefficient were used to examine the internal consistency of the SPAIB-P. Age and gender differences were tested by means of ANOVA. Construct validity was examined by calculating Pearson product-moment correlation coefficients. Correlation coefficients between 0.10 and 0.29 are indicative of a weak association, between 0.30 and 0.49 of a moderate association, and 0.50 or higher of a strong association (Cohen, 1988). To examine whether gender moderated the degree of adolescent-parent agreement, additional correlations between adolescent and parent reports were computed for boys and girls.

To obtain evidence of the construct validity of the instrument, we tested the original unidimensional model proposed by the original study using the confirmatory factor analysis (CFA) procedure. Analyses were conducted using the robust maximum likelihood (robust ML) method. The following indices were reported: Satorra Bentler’s chi-square (S-B χ2), robust root mean square error approximation (RMSEA), comparative fit index (CFI), non-normalized fit index (NNFI). For RMSEA, values below 0.05 indicate a good fitting model and below 0.008 acceptable (Schumacker and Lomax, 2004). CFI and NNFI values indicate a good fit with values greater than or equal to 0.95 and an acceptable fit when greater than or equal to 0.90 (Bentler, 1990). The factorial invariance of the model (FI) was analyzed following the procedure suggested by Byrne (2006), according to which measurement invariance applies to) the base model, equivalence of factor loadings, the equivalence of intercepts, and equivalence of variance errors. According to the methodology proposed by Cheung and Rensvold (2002), we report the CFI, ΔCFI, ΔNNNFI, and ΔRMSEA. For ΔCFI increments less than or equal to −0.01 indicate that the null hypothesis of invariance should not be rejected; for the rest, the critical values are −0.001.

## Results

### Internal Consistency

Internal consistency (Cronbach’s alpha) of the SPAIB-P scale was found to be 0.98, the glb was 0.99, and the omega total coefficient was 0.98 (CI, 95%; 0.92–0.99).

### Gender and Age Differences

SPAIB-P mean was 21.02 (*SD* = 17.74). The ANOVA showed that parents did not differ significantly in SPAIB-P scores provided for their boys (*M* = 20.24; *SD* = 15.18) and girls (*M* = 21.38; *SD* = 18.87) (*p* > 0.05). No significant main effects of age or interaction effects of gender and age were found.

### Construct Validity

Pearson product-moment correlations were computed between the SPAIB-P score and conceptually related social anxiety measures answered by adolescents and parents. The SPAIB-P correlated strongly with the FNE, SAD-N, SAD-G subscales, and Total score of the SAS-P (0.81, 0.90, 0.87, and 0.90, respectively). These high correlations (above 0.50) suggest that these parent versions of SPAIB and SAS-P scales are highly correlated. The correlation coefficients were statistically significant in all cases (*p* < 0.01). However, correlations between SPAIB-P and children-reported social anxiety measures, such as the SPAI-B and the FNE, SAD-N subscales, and Total score of the SAS-P were weak and non-significant (0.04, 0.05, 0.02, 0.03, and 0.05, respectively). Based on gender, parent-youth correlations were −0.10 for boys and 0.13 for girls. Agreement was significantly higher for girls (*p* < 0.01). It must be noted that the highest level of agreement was obtained from one of the most observable Item 10 (SAD-N subscale), “My child get nervous when s/he talks to peers s/he doesn’t know very well” to the lowest agreement in item 16 (filler item).

### Confirmatory Factor Analysis

We employed robust maximum likelihood estimators (MLM) for the model parameters. All items in CFA were loaded 0.75 or greater, ranging between 0.77 (item 9) and 0.82 (item 2). According to the results, the one-factor model fit the data very well: χ2/df ratio = 200.894, RMSEA = 0.80, SRMR, CFI = 0.97, and NNFI = 0.97.

### Testing Measurement Invariance (Gender and Age)

According to Table 1, data related to gender revealed the adequacy of the model fit to data (configural invariance), with CFI = 0.97 and RMSEA = 0.09. Furthermore, the ΔCFI between this model and the baseline 1 was < 0.01, below the cut point 0.01 proposed by Cheung and Rensvold (2002), so factor loadings were considered equivalent across gender (weak invariance). Finally, items were forced to be equal across gender groups (strong invariance). The ΔCFI with respect to the previous model was 0.004, so intercepts may also be considered equivalent across gender. Finally, the ΔCFI with respect to the previous model was 0.010, so there was equivalence of residuals of items, concluding total invariance.

**TABLE 1:** CFA: Gender and age invariance.

**1.1.CFA and *gender* invariance**									
	**Overall Fit Indices**					**Comparative Fit Indices**			
	**S/B χ 2**	**df**	**CFI**	**NNFI**	**RMSEA [90% CI]**	**Model comparison**	**Δ CFI**	**Δ NNFI**	**Δ RMSEA**
**Baseline**									
Unidimensional structure	200.89	104	0.97	0.97	0.08 [0.06–0.10]	–			
Boys	153.33	104	0.94	0.93	0.10 [0.06–0.13]				
Girls	179.06	104	0.98	0.97	0.08 [0.06–0.11]				

***Multigroup measurement invariance (sex)***									
1. Configural	333.27	240	0.97	0.96	0.09 [0.07–.011]				
2. Metric (weak invariance)	355.09	223	0.97	0.96	0.09 [0.07–0.11]	1 vs. 2	–0.002	0	–0.001
3. Scalar (strong invariance)	377.6	239	0.96	0.96	0.09 [0.07–0.10]	2 vs. 3	–0.004	–0.007	–0.001
4. Total (strict/invariant uniqueness)	366.65	255	0.97	0.97	0.08 [0.06–0.10]	3 vs. 4	0.01	0.012	–0.006

**1.2.CFA and *age* invariance (12–14year vs. 15–18 year)**									
	**Overall Fit Indices**					**Comparative Fit Indices**			
	**SB χ 2**	**df**	**CFI**	**NNFI**	**RMSEA [90% CI]**	**Model comparison**	**Δ CFI**	**Δ NNFI**	**Δ RMSEA**

**Baseline**									
Unidimensional structure	200.89	104	0.97	0.97	0.08 [0.06–0.10]				
12–14	164.33	104	0.97	0.96	0.09 [0.06–0.11]				
15–18	159.71	104	0.97	0.97	0.09 [0.06–0.11]				

***Multigroup measurement invariance (sex)***									
1. Configural	319.48	206	0.97	0.97	0.09 [0.07–0.10]				
2. Metric (weak factorial)	335.94	221	0.97	0.97	0.08 [0.07–0.10]	1 vs. 2	0	0.002	–0.002
3. Scalar (strong factorial)	385.2	255	0.97	0.96	0.09 [0.07–0.10]	2 vs. 3	–0.005	–0.008	0.004
4. Total (strict/invariant uniqueness)	370.69	256	0.96	0.96	0.09 [0.07–0.11]	3 vs. 4	–0.002	–0.002	0.004

*Models: 1. Configural invariance (M1, baseline model; equivalence of model form); 2. Metric invariance (equivalence of factor loadings), M2: M1+ factor loadings; 3. Scalar invariance (equivalence of item intercepts or thresholds), M2+ intercepts, and (d) 4. Total or residual invariance (equivalence of items’ residuals or unique variances) (M4: M3+ items’ residuals).*

Similarly, analyses for age were conducted to determine whether the unidimensional structure of SPAIB-parent form was invariant across two age groups: middle adolescents (12–14 years old) and late adolescents (15-18 years old). CFI = 0.97 and RMSEA = 0.08 reflected an adequate fit of the baseline model (configural invariance). Then, weak, strong, and total invariance hypotheses were accepted, since ΔCFI was lower than 0.010 in all comparisons.

## Discussion

Our objective was to examine a brief, valid, and reliable parent version administered *via* Internet of a well-established social anxiety child measure. Our data confirmed SPAIB-P score was not significantly different between adolescent and parent reports: SPAIB score was 29.43 (*SD* = 10.70) based on an adolescent report, and 21.02 (*SD* = 17.74) based on SPAIB-P. In addition, parents were reliable for identifying the social anxiety symptomatology of their children, but a higher agreement was found for girls. This is consistent with authors who also found parents can be reliable reporters of social anxiety of their adolescents (La Greca et al., 2001), with higher parent-teen correlations for girls (Perez et al., 2001; Garcia-Lopez et al., 2010). As far as the correspondence between the adolescent and parent reports is concerned, an interrater agreement was low. This is also aligned with the work of Perez et al. (2001), who indicated low-to-medium agreement but lower than parent and child responses to anxiety measure (Runyon et al., 2018). Furthermore, confirmatory factor analysis of the parent-report version of the SPAIB-P yielded essentially the unifactorial structure as the adolescent report version (for a review, please see Garcia-Lopez et al., 2008).

Good measurement invariance was found across gender and age, consistent with child-version SPAIB (Olivares et al., 2004; Vieira et al., 2013). Thus, our findings demonstrate that the factor loading and factor invariances and covariances were equivalent across gender and age groups of Spanish adolescents. Therefore, it may be recommended administering SPAIB-P for identifying of social anxiety symptomatology of children. Multi-method and multi-informant are crucial. This parent version could be used as a complementary measure to avoid the overestimation of the social anxiety symptomatology of his or her child.

### Limitations

Some limitations must be noted. Even though our study was strengthened by the use of a sample of adolescents with a primary clinical diagnosis of SAD according to DSM-5, the limited number of performance-only SAD-specifier adolescents is a drawback of this study, which might have affected findings. Similar to other studies, our sample was composed by a limited number (15%) of performance-specifier individuals (Burnstein et al., 2011; Kerns et al., 2013; Garcia-Lopez et al., 2016, 2018). Uniqueness of a performance-only specifier (lower social anxiety scores and comorbidity rates) appears to be consistent with findings from other researchers (Bögels et al., 2010; Garcia-Lopez et al., 2018), who have suggested that the specifier presents a different pattern than a full spectrum of SAD. Therefore, future studies should look at examining the discriminative validity of the SPAIB-P to differentiate the presence of specifier in their children. In addition, it is crucial to examine if our results generalize to different cultures, community samples, and paper-and-pencil format. As a sample was composed by socially anxious adolescents and no control condition was available, future studies should focus on providing cut-off scores. Finally, the high-consistency value suggests future studies could explore the reduction of the number of items by eliminating redundancy or poorly performing items.

Although efficacious treatments for pediatric SAD are available, underdiagnosis is associated with undertreatment and poorer outcomes. Due to difficulties in detecting mental health emotional disorders in their early stages and overestimation of adolescents of their symptomatology, it is crucial to complement it with our resources. Parents may provide useful information about their perception of the social anxiety levels of their children. Our findings suggest this parent-based measure provided similar information for their children who are clinically diagnosed with social anxiety.

## Data Availability Statement

The raw data supporting the conclusions of this article will be made available by the authors, without undue reservation.

## Ethics Statement

The studies involving human participants were reviewed and approved by Bioethical Commitee, University of Jaen. Written informed consent to participate in this study was provided by the participants’ legal guardian/next of kin.

## Author Contributions

L-JG-L contributed to data collection and data analysis and wrote the manuscript. LE-F coordinated data collection and wrote the manuscript. J-AM-M contributed to data collection and manuscript revision and wrote part of the results section. JP performed part of the statistical analysis, wrote part of the results section, and contributed to manuscript revision. All authors contributed to the article and approved the submitted version.

## Conflict of Interest

The authors declare that the research was conducted in the absence of any commercial or financial relationships that could be construed as a potential conflict of interest.

## Publisher’s Note

All claims expressed in this article are solely those of the authors and do not necessarily represent those of their affiliated organizations, or those of the publisher, the editors and the reviewers. Any product that may be evaluated in this article, or claim that may be made by its manufacturer, is not guaranteed or endorsed by the publisher.
